# Search model based on Kalman Filter and Monte Carlo simulation

**DOI:** 10.1371/journal.pone.0339117

**Published:** 2026-02-13

**Authors:** Jinhan Liu, Yujing Li, Xuhua Liu

**Affiliations:** College of Science, China Agricultural University, Beijing, Beijing, China; University of Southern California, UNITED STATES OF AMERICA

## Abstract

In the search and rescue operation of the submersible, to better search for the missing or faulty submersible, taking the marine environment simulated by the HYCOM model as the sample, it is necessary to use the Kalman filter model to predict the time location of the submersible and provide information support for the follow-up search and rescue operations according to the position information transmitted to the main ship when the submersible is running normally. Monte Carlo simulation is used to quantitatively analyze the probability of the possible area of the submersible in four possible cases after the fault, to obtain the location of the initial search deployment point, that is, the minimum plane projection area of the covering sample. Python software was used to quantitatively analyze the probability of finding the submersible with the passage of time and cumulative search results. Moreover, we conducted a comparative analysis of the method proposed in this paper with previous methods to illustrate the advancement of the method proposed in this paper. By introducing the nearest neighbor correlation algorithm into the multi-target tracking algorithm, the motion position of multiple submersibles in the same area can be predicted.

## 1 Introduction

In recent years, as mankind’s passion for exploring the unknown in the depths of the oceans has increased, submersibles have become an important tool for exploring this mysterious realm. However, the complexity and unpredictability of the deep sea environment pose great challenges to the safe operation of submersibles. Submersibles may encounter a variety of unexpected situations during their missions, such as loss of communication with the main vessel and mechanical failures, which greatly increase the risk of diving expeditions. Therefore, to better assist in the search and rescue of malfunctioning or missing submersibles, we should develop a model that can predict the changing position of the submersible over time.

In the field of search and rescue of submersibles, positioning has always been a top priority. Numerous studies have proposed various localization and search methods. For example, Kalman filtering, as an effective prediction and estimation technique, has been widely used in the prediction of submersible localization.Pradlwarter HJ and Schuëller GI [1] develop a local domain Monte Carlo framework enhancing probabilistic simulation accuracy while exploring failure domains.Fleming CH, Sheldon D, Gurarie E, Fagan WF, LaPoint S and Calabrese JM [2] propose a “local domain Monte Carlo simulation” integrating Kalman filtering with localized sampling, achieving substantial computational efficiency gains. This method dynamically identifies high-probability regions in state spaces for continuous animal movement models, balancing nonlinear adaptability and computational efficiency through localized linearization priors and adaptive sampling domains. It demonstrates utility in behavioral analysis and missing data imputation for large ecological datasets. Peng X, Zhang B and Rong L [3] achieve breakthroughs with a Modified Unscented Kalman Filter (MUKF) incorporating chi-square anomaly detection and dynamic noise covariance adjustment, meeting real-time motion requirements. Lu JY and Li X [4] propose a Kalman filter algorithm to estimate the velocity and direction of an indoor robot, employing RSSI-based localization to obtain positional coordinates. Luo Q, Yan X, Ju C, Chen Y and Luo Z [5] introduce an adaptive Kalman filter-based Ultra-Short Baseline (USBL) positioning system that significantly improves localization accuracy through real-time covariance matrix adjustment by Autonomous Underwater Vehicles (AUVs). Zhang J and Zhu Y-Z [6] employ the AP98 wind-pressure drift model to simulate maritime drowning victims, utilizing Monte Carlo methods to predict floating trajectories. Ming Li, Xiafei Tang, Qichun Zhang and Yiqun Zou [7] propose a Gaussian mixture noise-based Pseudo-Linear Minimum Mean Square Error (PL-MMSE) filter for azimuth Target Motion Analysis (TMA), integrating state constraints. Cao X and Yu A-L [8] propose a Self-Organizing Map (SOM) and Growing Neural Gas Network (GNGN) fusion algorithm, validated for superior target search performance in complex marine environments.Zhang Y, Yue S, Xu K, Zhang Z, Zhou L, Zhang Y and Lü G [9] conduct cross-validation using Argo float data and HYCOM simulations, revealing spatiotemporal error patterns in global ocean current models. This work optimizes parameterization schemes and enables paradigm shifts from surface observations to 3D current field corrections. Qichun Zhang , Zhengtao Ding and Hong Wang [10] introduce an information-theoretic minimum entropy filtering framework for single-output nonlinear systems, combining entropy minimization with reduced-order observer structures to advance nonlinear filtering methodologies.

However, most existing research focuses on technical optimizations, with limited exploration of integrated emergency response strategies for submarine crash incidents. Particularly in complex marine environments, the development of scientifically rigorous and operationally feasible search-and-rescue models - through integration of real-time submersible data with ocean dynamic models - remains a critical unresolved challenge. Against this research backdrop, the present study aims to build upon existing findings to deliver systematic solutions for submarine localization, trajectory prediction, and practical search-and-rescue operations.

## 2 Materials and methods

To better focus on the essence of the problem and model development, the following assumptions are made:

During normal operation, the submersible’s propulsion system maintains an approximately uniform motion. Since sudden acceleration or deceleration may introduce prediction deviations, this study concentrates on position estimation under steady-state motion. Based on the quasi-constant velocity assumption, the control input *u*_*t*_ in the Kalman filter is set to zero.

It is also assumed that the submersible transmits its position to the mother ship every 5 seconds. This study exclusively considers submersibles connected to the mother ship.

### 2.1 HYCOM

The HYCOM model utilizes a triple hybrid vertical coordinate system, designed to dynamically adapt coordinate types according to water depth in order to enhance numerical precision.

In the ocean surface layer, which spans from 0 to 100 meters below sea level, the HYCOM model utilizes a fixed-depth Z-coordinate system to examine the dynamics of wind-driven mixed layers. Surface forcing processes are applied via drifters that track ocean currents. The core equations are as follows:


Horizontal momentum equation:

$$\frac{\partial u}{\partial t}+u\cdot \nabla u+fk\times u=-\frac{1}{\rho_{0}}\nabla p+\frac{\partial }{\partial z}\left(\nu_{v}\frac{\partial u}{\partial z}\right)+\frac{\tau_{\text{wind}}}{\rho_{0}\Delta z}$$ (1)

Mixed layer depth equation:

$$h_{\text{MLD}}=\kappa \frac{u_{*}^{3}}{g\alpha Q_{\text{net}}}$$ (2)

In the surface layer dynamics, the horizontal velocity vector *u* (m/s) characterizes three-dimensional seawater motion, influenced by the Coriolis parameter f=2Ωsinϕ (s^−1^, where Ω is Earth’s angular velocity), driven through the pressure gradient term ∇p (Pa) and wind stress $\tau_{\text{wind}}=\rho_{a}C_{d}\|U_{10}\|U_{10}$ (N/m^2^, containing 10m wind velocity *U*_10_). The turbulent mixing processes are governed by the vertical eddy viscosity coefficient $\nu_{v}$ (m^2^/s) and mixed layer depth $h_{\text{MLD}}=\kappa u_{*}^{3}/(g\alpha Q_{\text{net}})$ (m, incorporating friction velocity u_*_, thermal expansion coefficient *α*, and net heat flux $Q_{\text{net}}$).

In the mesopelagic zone of the ocean, ranging from 100 to 2000 meters below sea level, the model transitions to an isopycnal coordinate system. This shift minimizes errors associated with density advection, thereby improving the representation of mesoscale eddies and frontal structures, while also accounting for drifters positioned at neutral buoyancy points. The core equations are given below:


Potential vorticity conservation:

$$\frac{D}{Dt}\left(\frac{\omega_{a}\cdot \nabla \rho }{\rho }\right)=0$$ (3)

Quasi-geostrophic vorticity equation:

$$\frac{\partial \zeta }{\partial t}+\beta v=\frac{f_{0}}{\rho_{0}}\frac{\partial }{\partial z}\left(\frac{w_{e}}{N^{2}}\right)$$ (4)

Density advection equation:

$$\frac{\partial \rho }{\partial t}+u\cdot \nabla \rho =\kappa_{h}\nabla_{h}^{2}\rho +\frac{\partial }{\partial z}\left(\kappa_{v}\frac{\partial \rho }{\partial z}\right)$$ (5)

In the intermediate layer , the potential vorticity conservation equation couples absolute vorticity $\omega_{a}=\nabla \;\times \;u\;+\;2\Omega$ (s^−1^) with density field *ρ* (kg/m^3^), while the quasi-geostrophic vorticity equation resolves mesoscale eddy evolution through relative vorticity ζ (s^−1^), beta-plane parameter β=2Ωcosϕ/R (m^−1^s^−1^), and buoyancy frequency $N^{2}=-g\rho_{0}^{-1}\partial \rho /\partial z$ (s^−2^). Density advection processes are regulated by horizontal/vertical diffusion coefficients $\kappa_{h}$ (m^2^/s) and $\kappa_{v}$ (m^2^/s).

In the ocean bottom layer at depths exceeding 2000 m below sea level, the HYCOM model utilizes a terrain-following *σ* coordinate system. This approach dynamically adjusts layer thickness to match seabed topography, allowing for accurate representation of turbulent mixing within the bottom boundary layer. It also supports modeling of drifters that have settled onto the seafloor. The core equations are as follows:


Topography-adjusted momentum equation:

$$\frac{\partial u}{\partial t}+u\cdot \nabla u+fk\times u=-\frac{1}{\rho_{0}}\nabla p-g\nabla \eta +\nabla \cdot (\nu_{h}\nabla_{h}u)+\frac{\partial }{\partial \sigma }\left(\nu_{v}\frac{\partial u}{\partial \sigma }\right)$$ (6)

Continuity equation:

$$\frac{\partial \eta }{\partial t}+\nabla \cdot \left[(H+\eta )u\right]=0$$ (7)

Turbulent kinetic energy equation:

$$\frac{\partial k}{\partial t}=\nu_{v}{\left(\frac{\partial u}{\partial z}\right)}^{2}-\epsilon +\frac{\partial }{\partial z}\left(\nu_{k}\frac{\partial k}{\partial z}\right)$$ (8)

$$\sigma =\frac{z-\eta }{H+\eta }$$

In the abyssal layer , the terrain-following *σ*-coordinate σ=(z−η)/(H+η) (dimensionless) adapts to bottom topography, with free surface height *η* (m) and resting depth *H* (m) connected through continuity equation. The turbulent kinetic energy *k* (m^2^/s^2^) equation quantifies bottom boundary layer mixing intensity, incorporating dissipation rate *ε* (m^2^/s^3^) and diffusion coefficient $\nu_{k}$ (m^2^/s). All equations employ reference density $\rho_{0}$ (≈1025 kg/m^3^) and gravitational acceleration *g* = 9.81 m/s^2^ as fundamental constants.

Taking the HYCOM virtual ocean environment as the starting points, we use Python to generate random points within an 80-nautical-mile radius around each predicted drifter location—simulated at 3-hour intervals over the 72 hours following the incident. This approach models potential positional errors and drifting trajectories under disturbance, forming a sample set for further analysis.

### 2.2 Kalman Filter model

The Kalman filter is an algorithm designed for optimal state estimation of linear systems, using a series of measurements observed over time that contain statistical noise. It operates under conditions of known measurement noise covariance to recursively estimate the system state. The filter also supports real-time updating of on-site acquired data. In positioning applications such as submersible navigation, monitoring equipment can be configured to periodically transmit location information to a mother ship as known state data. Based on these received position updates, the Kalman filter predicts the submersible’s location during 5-second communication gaps, thereby constructing a mathematical model that continuously forecasts position over time.

To improve the application of the Kalman filter for position prediction, the three-dimensional space in which the submersible operates can be decomposed into three one-dimensional spaces along the X, Y, and Z axes, each analyzed separately. This allows for independent state estimation per spatial component. Beginning with the X-axis: Here, *p*_*t*_ represents submarine position, $v_{t}$ represents submarine speed, *u*_*t*_ represents control quantity, $\hat{x}$ represents estimating the state of a submersible,*σ* represents the variance of two dimensions, F represents state transition matrix, Z represents observation status, u represents observation matrix, *υ* represents observation noise, *K*_*t*_ represents Kalman coefficient.

Let’s assume that a set of position-state information transmitted from the submersible to the main ship is

$$x_{t-1}=(\begin{array}{c} p_{t-1}\\ v_{t-1} \end{array})$$ (9)

and the predicted position state of the submersible in the next second is

$$x_{t}=(\begin{array}{c} p_{t}\\ v_{t} \end{array})$$ (10)

where

$$p_{t}=p_{t-1}+v_{t-1}\times △t+u_{t}\times \frac{△t^{2}}{2},v_{t}=v_{t-1}+u_{t}\times △t$$ (11)

Therefore

$$(\begin{array}{c} p_{t}\\ v_{t} \end{array})=(\begin{array}{cc} 1 & △t\\ 0 & 1 \end{array})(\begin{array}{c} p_{t-1}\\ v_{t-1} \end{array})+(\begin{array}{c} \frac{△t^{2}}{2}\\ △t \end{array})u_{t}$$ (12)

We let

$$F_{t}=(\begin{array}{cc} 1 & △t\\ 0 & 1 \end{array}),B_{t}=(\begin{array}{c} \frac{△t^{2}}{2}\\ △t \end{array})$$ (13)

Then the previous equation can be simplified to

$$\hat{x_{t}^{-}}=F_{t}\hat{x_{t-1}^{+}}+B_{t}u_{t}$$ (14)

However, given the autonomous control capabilities of the submersible, the control input *u*_*t*_ will be set to zero for the X-axis in the subsequent modeling and programming phases of this study. The same applies to the Y and Z axes.

When predicting the position of a submersible using the Kalman filter, several sources of uncertainty affect the estimation accuracy. The noise primarily originates from two aspects: potential mechanical failures of the submersible itself, and inaccuracies in positional data. Abnormal currents or variations in water density may cause deviations when the submersible’s monitoring equipment transmits location information to the mother ship. In addition, failures in the propulsion system can impair the vehicle’s ability to control its direction and speed. These factors introduce noise that degrades the performance of the state estimation within the filter.

Thus, a covariance matrix is employed to quantify the estimation uncertainty of the submersible’s position. The location data received by the mother ship is treated as a set of distributed points in a three-dimensional coordinate system, which follows a Gaussian distribution along each axial direction, thereby allowing derivation of the corresponding covariance matrix to characterize the uncertainty.

$$cov(x,x)=(\begin{array}{cc} \sigma_{11} & \sigma_{12}\\ \sigma_{21} & \sigma_{22} \end{array})$$ (15)

In the analysis of submersible position prediction, the state uncertainty at each timestep is described by the covariance matrix *P*. The evolution of the covariance matrix over time is captured by the following equation:

$$P_{t}^{-}=F_{t}P_{t-1}^{+}F_{t}^{T}+Q$$ (16)

The matrix Q represents the process noise covariance matrix, which models the uncertainty or noise inherent in the system dynamics. It captures disturbances that cannot be precisely described during state prediction. The intensity of noise in the state transition equation has been quantitatively characterized. In this study, the Q matrix is explicitly defined as a 6×6 diagonal matrix with all diagonal elements set to 0.0001. This formulation describes the covariance relationships across different time instances. The observation matrix H is constructed as follows:

$$H=(\begin{array}{cc} 1 & 0 \end{array})$$ (17)

The relationship between the position *Z* of the submersible and the position state *X* is

$$z_{t}=Hx_{t}+v$$ (18)

where v is the noise of the observation. Let *R* be the covariance matrix of the observation noise. After obtaining the position *Z*, we can correct our prediction about the position of the submersible, i.e.,

$$\hat{x_{t}^{+}}=\hat{x_{t}^{-}}+K_{t}(z_{t}-H\hat{x_{t}^{-}})$$ (19)

where the Kalman coefficients are

$$K_{t}=P_{t}^{-}H^{T}(HP_{t}^{-}H^{T}+R{)}^{-1}$$ (20)

The matrix R is defined as a 3×3 identity matrix, with diagonal elements set to 1 and all off-diagonal elements being 0.

In the filter’s next iteration, the noise covariance matrix will be calibrated to improve estimation accuracy.

$$P_{t}^{+}=(I-K_{t}H)P_{t}^{-}$$ (21)

**Fig 1:**
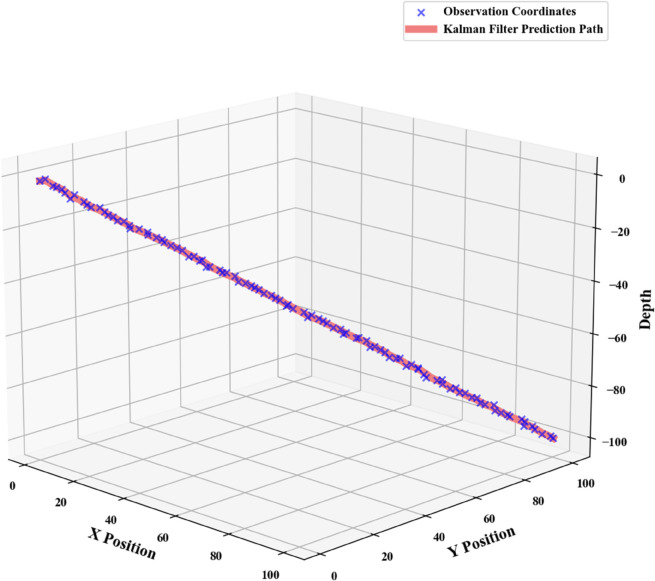
Kalman Filter prediction plot for submersible position.

Subsequent iterative computation using Python was conducted to predict the trajectory of the submersible. Fig 1 displays the corresponding position estimates generated by the Kalman filter algorithm.

### 2.3 Monte Carlo simulation model

This paper focuses on simulating communication and mechanical failures in submersibles and modeling search and rescue operations. We employ Monte Carlo simulation to perform probabilistic analysis of potential locations for disabled submersibles.

**Fig 2:**
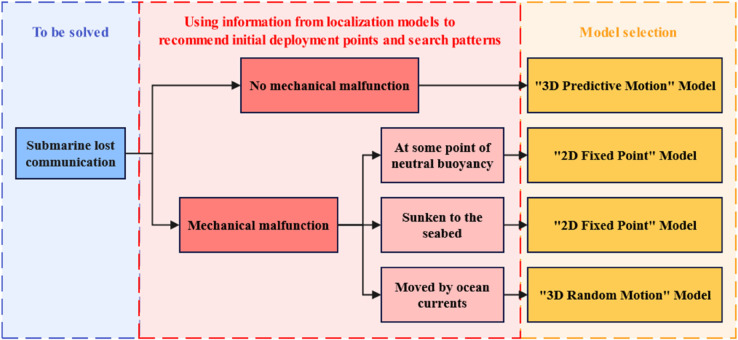
Faulty submersible condition classification chart.

When a submersible experiences only communication failure without mechanical failure, it remains operational. In such cases, trajectory prediction can be continued using the Kalman filter model from positioning systems to estimate future positions. When both communication and mechanical failures occur simultaneously, the submersible’s state is categorized into three operational modes: submerged on the seafloor, stationary at neutral buoyancy points, or drifting with ocean currents. These states are simplified for simulation purposes as: 2D random fixed points (seafloor), 3D random fixed points (neutral buoyancy), and 3D stochastic path points (current-driven drift). Monte Carlo simulations are conducted on these generated sample points to determine optimal search patterns and resource allocation. Leveraging positioning model data and search path optimization algorithms, this approach significantly reduces the time required to locate missing submersibles. The complete workflow is illustrated in Fig 2.

### 2.4 Algorithm architecture design

This study establishes a three-tiered hierarchical algorithmic framework within HYCOM-simulated marine environments, integrating Kalman filtering, Monte Carlo simulation, and path optimization. The core methodology proceeds as follows:

First, a Kalman filter incorporates multi-source observational data and dynamic models to achieve recursive estimation of the submersible’s three-dimensional motion state, while outputting the means and covariance matrices of trajectory predictions.

Second, Monte Carlo methods generate stochastic samples consistent with state distributions, emulating underwater environmental noise, sensor errors, and target motion randomness to form a stochastic trajectory ensemble.

Third, to identify optimal search patterns, multiple spatial scanning strategies—including spiral, parallel-line, sector-based, and expanding rectangular search modes—were developed. The effectiveness of each strategy is evaluated by integrating detection probability models to determine superior search paths.

With regard to the spiral search pattern, the fundamental parametric trajectory equations are as follows:

Parametric trajectory equation


$$\left\{ \begin{array}{c} x=r(t)\cos(\theta (t))+at\\ y=r(t)\sin(\theta (t))+at\\ z=z_{0}+\beta t \end{array}\right.\;\left\{ \begin{array}{c} r(t)=r_{0}+\gamma t\\ \theta (t)=\theta_{0}+\omega t \end{array}\right.$$

In this process, r(t) increases linearly over time while θ(t) grows in arithmetic progression, achieving an optimal balance between spatial coverage and operational efficiency.

For the parallel-line search pattern, the methodology is structured around three independent components: reciprocating scanning along the X-axis, progressive advancement along the Y-axis, and synchronized descent along the Z-axis. The X-axis scanning follows a sawtooth wave trajectory to ensure comprehensive coverage of the horizontal plane. Upon completion of each X-axis cycle, the Y-axis advances incrementally to avoid overlapping coverage. The Z-axis descent is synchronized with the movement of the submersible, enabling complete three-dimensional spatial coverage.

The sector-based search operates in polar coordinates, where coverage is expanded by adjusting the angular parameter *θ* and the radial parameter r.

The expanding rectangular search adopts a layered expansion strategy, which systematically broadens the scanning range and employs trajectory planning to achieve large-area coverage.

After completing the modeling of these four search patterns, this study conducted a comparative analysis of the detection probability values across different patterns under identical parameter configurations, with the specific experimental parameters detailed in the accompanying Table 1. The search pattern yielding the highest probability value was ultimately The pattern demonstrating the highest probability was selected as the optimal strategy. The implementation using Python code generated results that validated the final selection, as presented below Table 2.

**Table 1:** Parameter configuration table.

Parameter Name	Parameter Value	Description
initial_state	(0, 0, 0, 0, –10, 0)	Initial position and velocity vector
num_simulations	10,000	Number of simulation runs
time_steps	50	Temporal discretization steps
detection_radius	5.0	Detection range threshold
depth_range_lower	–200	Lower bound of operational depth
depth_range_upper	–10	Upper bound of operational depth
search_radius	80	Maximum search coverage radius
layers	3	Hierarchical search layers
direction	(1, 1, –1)	Search direction vector components

**Table 2:** Detection probability comparison.

Spiral:	78.3%
Parallel	72.1%
Sector	65.4%
ExpandingRect	68.9%

The data presented in the table indicate that the spiral search pattern exhibits the highest search probability, leading us to conclude that it is the optimal mode of search.

Traditional methods predominantly utilize Kalman filtering or particle filtering for state estimation, or depend exclusively on Monte Carlo simulation to generate stochastic trajectories. Such approaches often lack a structured, multi-stage framework capable of integrating multi-source data and addressing uncertainties across multiple dimensions. Moreover, conventional techniques tend to prioritize shortest-path planning or obstacle avoidance in static settings, overlooking the dynamically evolving probability distributions associated with target motion.

In contrast, the methodology proposed in this study employs the HYCOM model to simulate the marine environment and integrates dynamic modeling with observational data to produce state estimates and covariance metrics. This enables explicit quantification of both predictability and uncertainty in trajectory forecasting. Additionally, by sampling from the probability distributions derived via Kalman filtering, the approach explicitly captures complex noise and stochastic characteristics unique to underwater settings. The result is a probabilistic trajectory set that moves beyond the conventional reliance on single deterministic paths. This set is subsequently incorporated into a unified framework combining multiple search patterns and detection probability models to facilitate dynamic optimal path planning. Using Monte Carlo sampling to generate probabilistic maps, the model reframes the path search task as an optimization problem aimed at maximizing detection probability—an formulation that closely aligns with the practical demands of real-world underwater search operations.

Single-method approaches such as Kalman Filtering (KF) and Particle Filtering (PF) primarily offer state estimation capabilities but do not explicitly support subsequent path planning. In contrast, the proposed model directly transforms state estimates into actionable search strategies through integrated Monte Carlo simulations and path search operations.

Algorithms such as Rapidly-exploring Random Trees (RRT*) focus on obstacle avoidance and path feasibility while overlooking the dynamic probability distribution of the target. The proposed framework, however, incorporates target motion uncertainty into the path generation process via Monte Carlo sampling, dynamically integrating uncertainties during trajectory formation with the core objective of maximizing probabilistic coverage.

To ensure impartial and reliable algorithm comparison, this study employed a consistent experimental setup using the same dataset for evaluation. A set of 200 Monte Carlo samples was generated from the state covariance to reduce sampling bias (parameters are listed in Table 3). Results indicate that the proposed KF-MC-PS method achieved a search probability of 76.5%, significantly outperforming other methods (Table 4) and effectively improving submersible search and rescue success rates. Building on this, the KF-MC-PS method was further applied to four typical submersible scenarios and compared with two other methods, enabling a comprehensive performance evaluation.

**Table 3:** Kalman filtering - Monte Carlo simulation - Path search (KF-MC-PS).

Process noise (q)	0.01
Observation noise (r)	0.1
Monte Carlo perturbation coefficient	0.3
Disturbance frequency	Every 5 steps
Number of nodes	300
The number of adjacent nodes	10
Maximum connection distance	5.0
Particle number	200
Initial noise	0.5
Resampling noise	0.1
Maximum number of iterations	1000
Step length	3.0

**Table 4:** Comparison table of search probabilities for different methods.

	Detection rate	Path length	Time-consuming
KF-MC-PS	76.50%	142.3	0.048s
PF	71.00%	139.8	0.085s
RRT*	58.50%	135.2	0.231s

#### 2.4.1 Loss of communication only.

**Fig 3:**
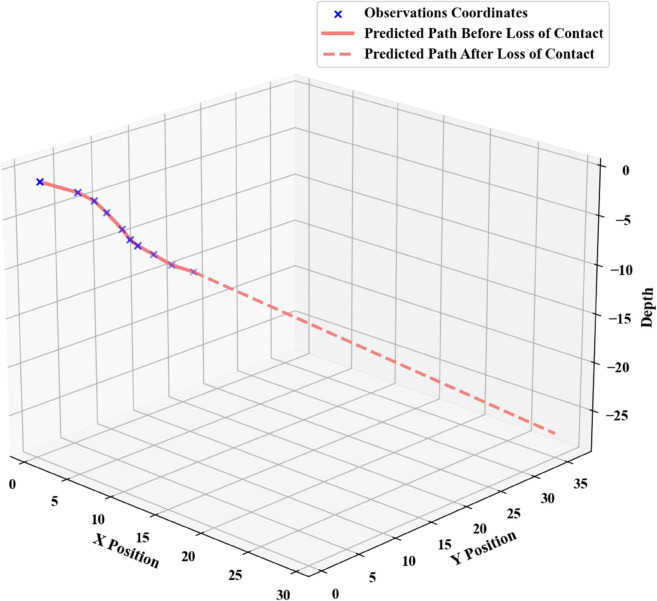
Charts of predicted submersible positions.

In scenarios where the submersible loses communication with the mother ship but remains fully operational, its trajectory must be predicted using the Kalman filter. Taking the last known position xx, transmitted prior to the loss of contact, as the initial state, the predictive update follows the equation: $\hat{x_{t}^{+}}=\hat{x_{t}^{-}}+K_{t}(z_{t}-H\hat{x_{t}^{-}})$. By executing the corresponding Python code, time-varying position estimates yy are obtained. Fig 3 illustrates a set of simulated trajectories under this prediction framework.

**Fig 4:**
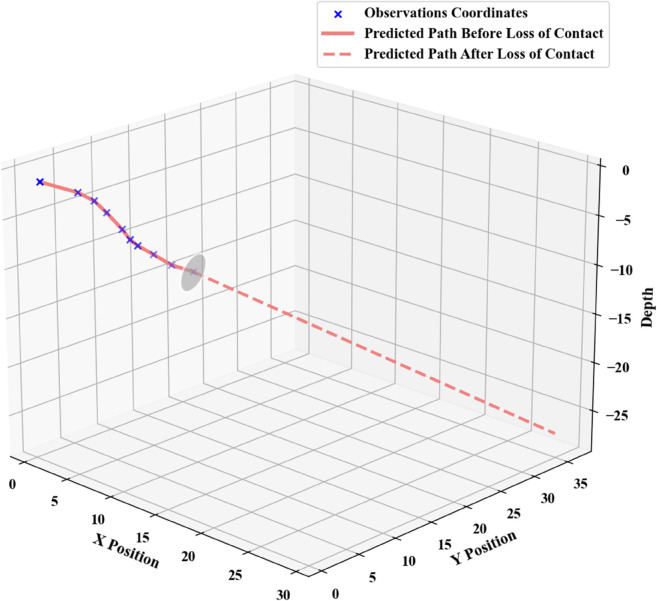
Initial deployment points.

Under such circumstances, the submersible’s position after a specified time horizon can be predicted, and this predicted location serves as the center for initial deployment. The specific deployment configuration is illustrated in Fig 4.

**Fig 5:**
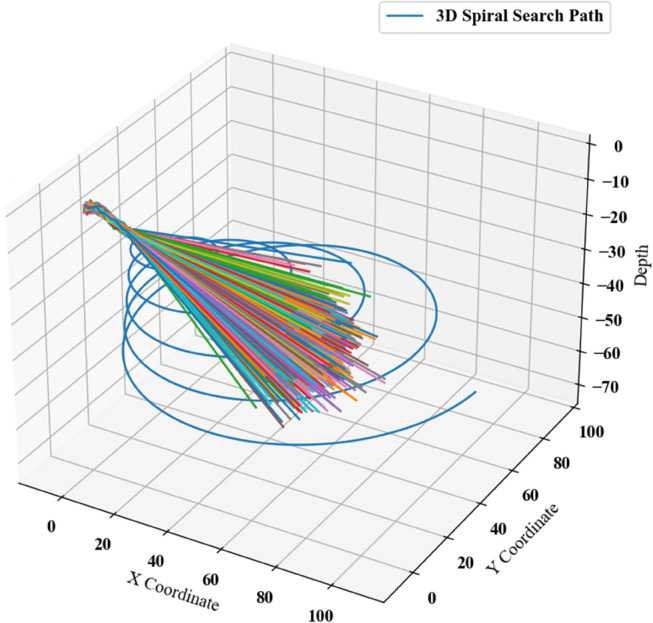
Spiral search for the first scenario.

Spiral search patterns were simulated in Python, starting from the predicted position and extending bilaterally along both sides of the estimated motion path. The simulation results are presented in Fig 5.

Subsequently, this study employs the Monte Carlo method via Python to compute the time-dependent detection probability and cumulative search results for the submersible. The focus lies on analyzing how temporal accumulation influences the detection probability: a location is considered successfully detected if the distance to the submersible is within a predefined detection radius.

In large-scale Monte Carlo simulations, the detection probability can be estimated as the frequency of successful detections within the detection radius—i.e., the ratio of the number of detected submersibles to the total number of simulated instances.

After establishing identical comparison conditions, the search probabilities of the proposed method (KF-MC-PS), PF, and RRT* for locating the submersible were compared. The cumulative detection probabilities over time yielded 53.06%, 6.55%, and 1.38% respectively. Corresponding parameter configurations are detailed in the Table 5 below.

**Table 5:** Table of parameter assignments for the first scenario.

q (process noise)	0.00025
r(Observation noise)	0.005
Monte Carlo noise scale	0.025
sample size	1100
detection radius	16.2

The Table 6 below presents comparative data for the three methods.

**Table 6:** Comparison of methods in the first case.

	Detection rate	Path length	Time-consuming
KF-MC-PS	53.06%	1000.2	82.589s
PF	6.55%	79.3	0.015s
RRT*	1.38%	80.9	0.063s

#### 2.4.2 The submersible has sunk to the seabed.

We now examine the case of mechanical failure of the submersible—specifically, the first scenario in which it has sunk to the seabed. As noted earlier, this situation can be modeled as a randomly generated two-dimensional fixed point. This simplification is based on two key considerations: the randomness of the final resting position after sinking, and the fact that its location becomes effectively independent of water depth.

**Fig 6:**
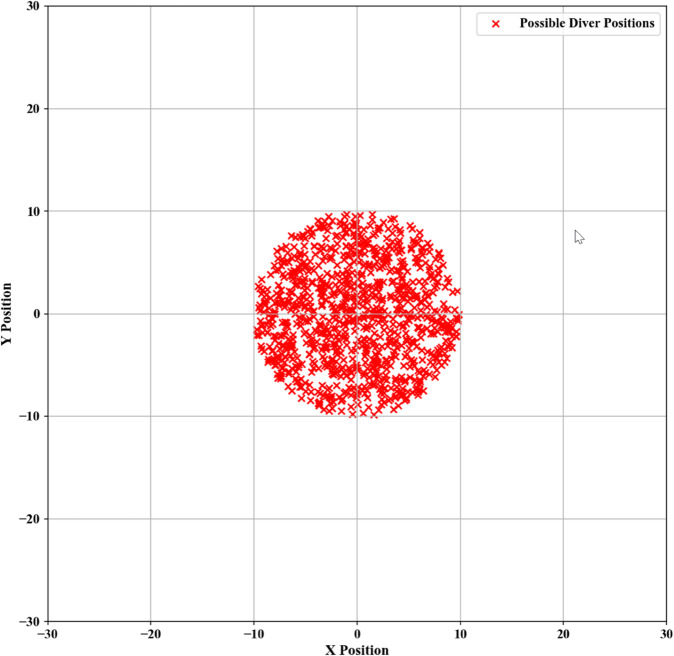
Charts of predicted submersible positions.

This study utilizes Python to generate stochastic three-dimensional models by using the last known position transmitted by the submersible before communication loss as the center point, and defining the radius based on the level of uncertainty. The generated configuration is presented in Fig 6.

**Fig 7:**
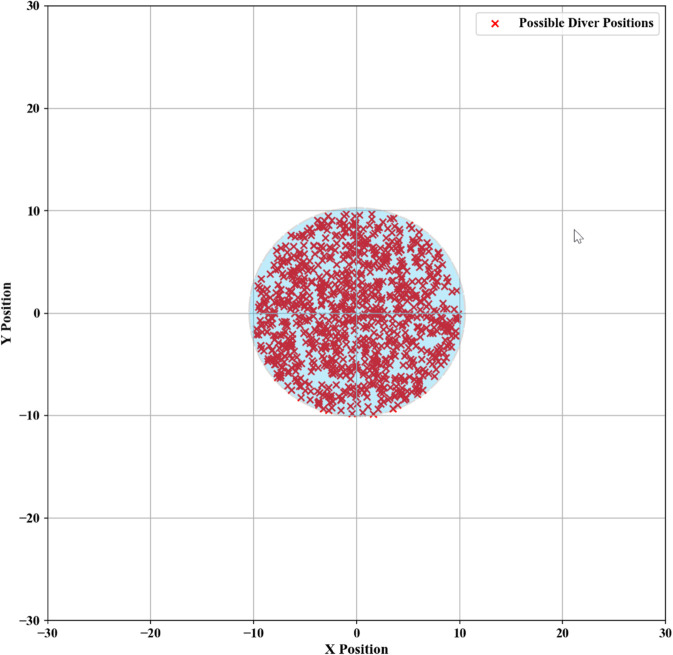
Initial deployment points.

The minimal region encompassing these randomly generated two-dimensional fixed points is identified as the optimal initial deployment location for search and rescue operations. The specific positional configuration is presented in Fig 7.

**Fig 8:**
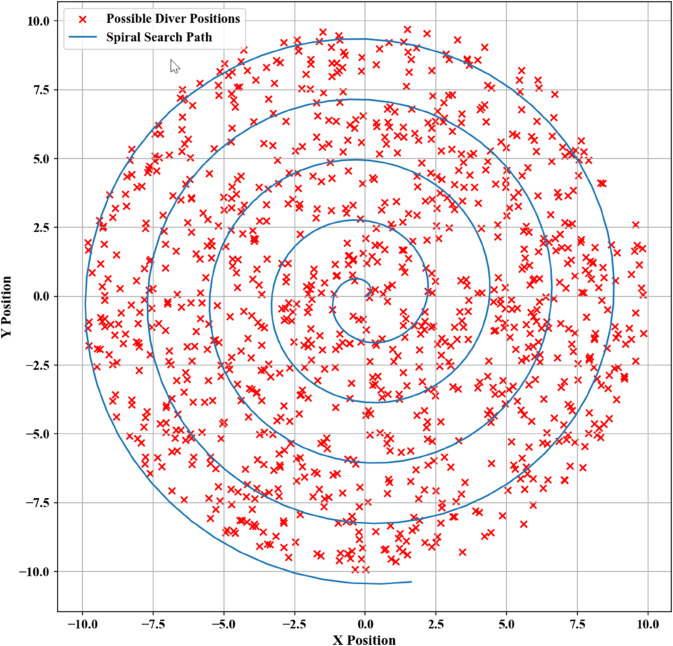
Spiral search for the second scenario.

Spiral search trajectories for this scenario were computationally generated using Python. The generated results are presented in Fig 8.

Python was subsequently applied to compute the probability of detecting the submersible over time and across cumulative search results, with particular emphasis on the influence of accumulated search effort on detection probability. The process began by calculating the distance between the submersible’s position and each point along the search path. Accordingly, the detection probability for this scenario is derived through the aforementioned Monte Carlo framework, yielding the result via the formula p=n/N.

Under identical comparison conditions, the search probabilities of the proposed method (KF-MC-PS), PF, and RRT* were compared. The cumulative detection probabilities over time for locating the submersible were 34.00%, 1.80%, and 1.80%, respectively. The corresponding parameter configurations are detailed in the Table 7 below.

**Table 7:** Table of parameter assignments for the second scenario.

q (process noise)	0.0002
r(Observation noise)	0.003
Monte Carlo noise scale	0.018
sample size	2800
detection radius	14.5

The Table 8 below presents comparative data for the three methods.

**Table 8:** Comparison of methods in the second case.

	Detection rate	Path length	Time-consuming
KF-MC-PS	34.00%	591.1	150.901s
PF	1.80%	15.0	0.000s
RRT*	1.80%	2.5	0.003s

#### 2.4.3 The submersible maintains position at the neutral buoyancy point.

**Fig 9:**
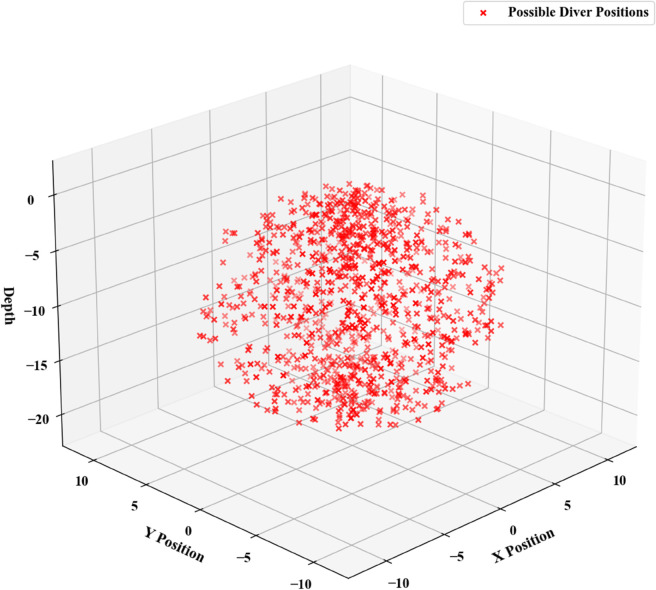
Charts of predicted submersible positions.

We now consider the scenario in which the submersible remains at a neutral buoyancy point. Under this condition, its average density equals that of the surrounding fluid, resulting in the cessation of vertical movement. This allows the situation to be modeled as a randomly generated three-dimensional fixed point. Using Python, the possible positions in 3D space were simulated via a spherical coordinate system: the last known position reported to the mother ship prior to the incident was set as the center of the sphere, with the polar angle 0≤Φ≤π and the azimuthal angle 0≤Φ≤π. Random points were then generated uniformly over the spherical surface, as shown in Fig 9.

**Fig 10:**
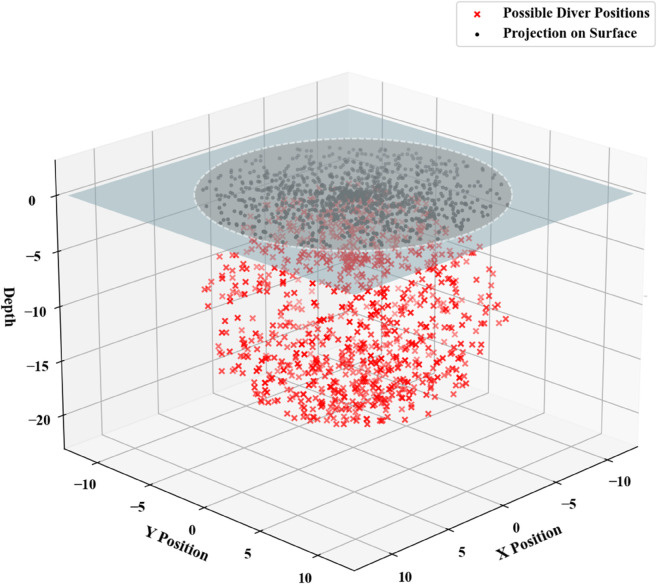
Initial deployment points.

The smallest enclosing projection in two dimensions containing the generated 3D fixed points is designated as the initial operational area for search and rescue missions. The deployment area is depicted in Fig 10.

**Fig 11:**
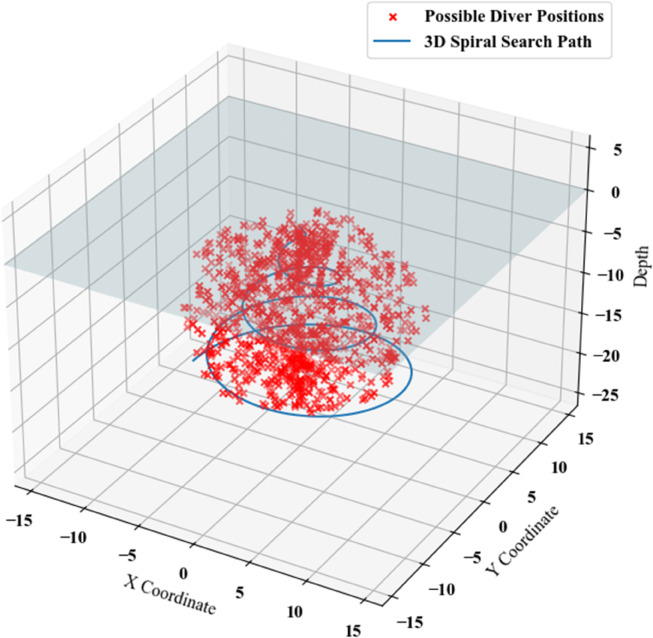
Spiral search for the third scenario.

Taking into account the depth coordinates of the 3D random points, a spiral search path was constructed by gradually varying the angular and radial parameters. This path was generated computationally using Python for the given context, producing the result shown in Fig 11.

Similarly to the case with two-dimensional fixed points, the Monte Carlo method was applied to estimate the detection probability of the submersible and its cumulative variation over time. This study focuses on examining how accumulated search results influence the detection probability: a location is considered detected if the distance from the search agent to the submersible is less than a predefined detection radius. The computational methodology for the detection probability in this scenario is analogous to that of the aforementioned cases.

Under identical experimental conditions, the search probabilities of the proposed KF-MC-PS method were compared with those of PF and RRT* algorithms. The cumulative detection probabilities for submersible localization over time were measured as 5.60%, 1.20%, and 1.90%, respectively. The corresponding parameter configurations are specified in the following Table 9.

**Table 9:** Table of parameter assignments for the third scenario.

q (process noise)	0.15
r(Observation noise)	1.5
Monte Carlo noise scale	3.0
sample size	15
detection radius	2.0

The Table 10 below presents the corresponding comparative data for the three methods.

**Table 10:** Comparison of methods in the third case.

	Detection rate	Path length	Time-consuming
KF-MC-PS	5.60%	670.9	0.852s
PF	1.20%	15.0	0.000s
RRT*	1.90%	12.6	0.008s

#### 2.4.4 The submersible drifted with ocean currents.

Finally, we consider the scenario in which the submersible is carried by ocean currents and other environmental factors. This situation can be modeled as a random walk of a point in three-dimensional space. Multiple stochastic trajectories are generated to simulate the drift behavior of the submersible, and detection probability estimates are conducted using the Monte Carlo method.

**Fig 12:**
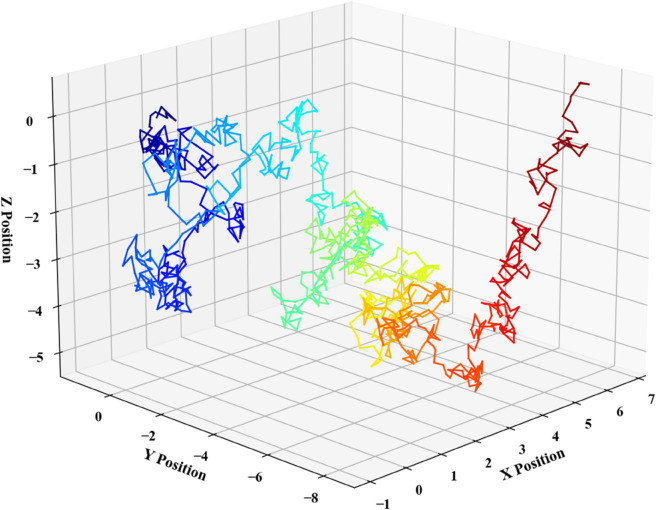
Charts of predicted submersible positions.

Using Python, the position arrays were initialized with randomly generated directions and step sizes, producing stochastic 3D motion trajectories as illustrated in Fig 12.

**Fig 13:**
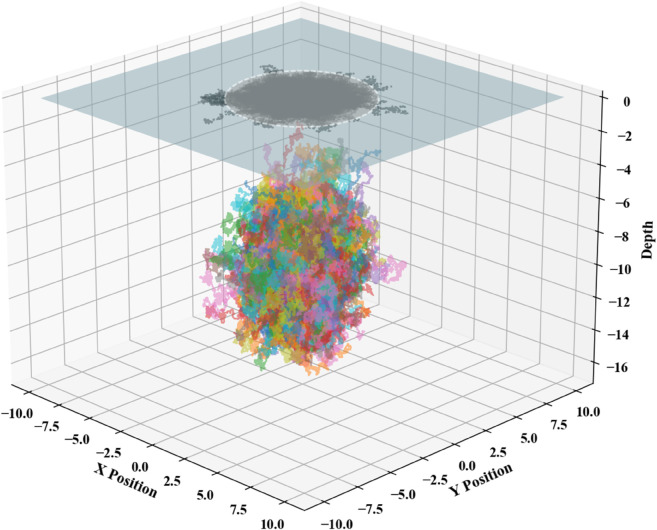
Initial deployment points.

The smallest projected area on the sea surface that encloses the generated three-dimensional stochastic paths can be designated as the recommended initial deployment zone for SAR (Synthetic Aperture Radar) operations, as shown in Fig 13.

**Fig 14:**
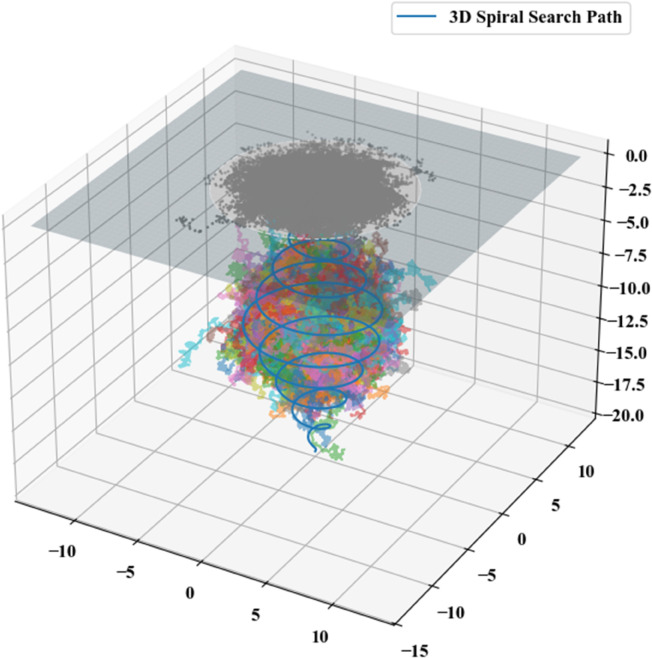
Spiral search for the fourth scenario.

Spiral search trajectories for this scenario were computationally generated via Python, producing the results illustrated in Fig 14.

Employing Monte Carlo simulations implemented in Python, this study computes the time-varying detection probability of the submersible and its cumulative search results, with a particular focus on the effect of temporal accumulation. A position is considered detected if the distance to the submersible is less than the specified detection radius. For a sufficiently large sample size, the detection probability is derived using Monte Carlo methods as the ratio of the number of detected submersibles to the total number of samples.

Under identical comparison conditions, the search probabilities of the proposed method (KF-MC-PS), PF, and RRT* were compared, yielding cumulative detection probabilities over time for locating the submersible of 25.42%, 23.02%, and 22.70%, respectively. The corresponding parameter configuration Table 11 is presented below.

**Table 11:** Table of parameter assignments for the fourth scenario.

q (process noise)	8.0
r(Observation noise)	18.0
Monte Carlo noise scale	22.0
sample size	10
detection radius	0.7

The Table 12 below presents comparative data for the three methods.

**Table 12:** Comparison of methods in the fourth case.

	Detection rate	Path length	Time-consuming
KF-MC-PS	25.42%	844.0	0.588s
PF	23.02%	3.0	0.011s
RRT*	22.70%	1.8	0.000s

### 2.5 Model extensions

#### 2.5.1 Model extension for the corresponding sea area.

The localization model utilizes a Kalman filter framework, which is prone to noise that compromises prediction accuracy. Subsea topography and ocean currents cause interference in submersible signals, leading to errors in the position coordinates received by the mother ship. These errors propagate as noise into the Kalman filter. Due to significant differences in seabed terrain and current-induced disturbances between complex and simple marine environments, the noise characteristics vary considerably. As a result, the covariance matrix parameters in the localization model must be dynamically adjusted in complex marine conditions to adapt to environment-specific noise profiles and ensure optimal performance.

The search model addresses four scenarios for a disabled submersible: (1) communication failure without mechanical damage, (2) sinking to the seabed, (3) maintaining neutral buoyancy, and (4) drifting with ocean currents. For scenarios 1 to 3, the localization model predicts the trajectory after communication loss, deploys initial search points at designated positions, and initiates spiral search patterns centered on the predicted locations. Throughout the process, localization parameters and error margins are adaptively adjusted based on marine environmental conditions while maintaining the search methodology unchanged. Scenario 4 is simplified as random motion in three-dimensional space.

#### 2.5.2 Model extensions on multiple submersibles moving in the same general area.

**Fig 15:**
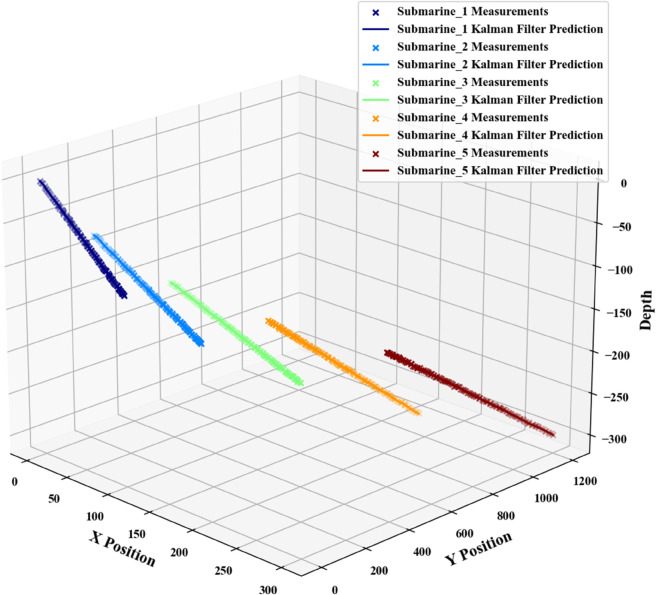
Prediction plot for multiple submersible movement paths.

To enhance applicability and achieve generalization, this study incorporates the Nearest Neighbor Association Algorithm to extend the Kalman Filter model. For multi-submersible tracking in overlapping regions, a multi-target tracking algorithm is applied, which resolves correlations between predicted position values to enable model expansion. Kalman filter parameters are initialized using Python, followed by the construction of the error covariance matrix, state transition matrix, process noise covariance matrix, measurement matrix, and measurement noise covariance matrix—all configured consistently within the localization problem framework. Data correlation analysis is performed iteratively: the initial cycle is skipped due to the absence of historical predictions, followed by multiple iterations based on correlation metrics. Upon completion of the iterations, Extended Kalman Filter(EKF) predictions are updated and visualized via Python simulation. With five submersibles configured, the results shown in Fig 15 are obtained.

## 3 Conclusions

### 3.1 Discussion

Traditional underwater vehicle trajectory prediction methods exhibit significant limitations in complex marine search-and-rescue scenarios. State estimation approaches like Kalman or particle filtering, coupled with Monte Carlo trajectory generation, struggle to integrate multi-source data and manage multidimensional uncertainties. Furthermore, their static path planning paradigms fail to capture dynamic oceanic motion characteristics, leading to substantial deviations between predicted and actual trajectories that compromise search accuracy and timeliness.

To address these challenges, this study introduces a novel dynamic prediction framework. By integrating HYCOM oceanographic models with real-time observations, the framework establishes a covariance-informed state estimation system that quantifies prediction uncertainties. Leveraging probability distribution samples generated through Kalman filtering, it effectively simulates underwater noise complexity while abandoning reliance on deterministic single-path models. Additionally, the framework innovatively couples multi-modal search strategies with detection probability models, enabling dynamic integration of state estimation and path planning for adaptive optimization. This approach significantly enhances operational adaptability in complex marine environments.

The proposed method offers two key advantages. First, its extensive data source compatibility allows predictions to be made using only standard operational data from the vehicle, eliminating the need for additional sensors and significantly reducing implementation costs. Second, the modular architecture ensures high scalability, enabling adaptation to diverse marine environments and multiple vehicle types. This research not only provides an efficient technical solution for underwater search-and-rescue operations but also establishes a new methodological paradigm for integrating dynamic probabilistic prediction with path planning.

Future work will focus on two directions. First, incorporating acoustic and visual SLAM technologies to improve state estimation accuracy. Second, developing cross-marine environmental transfer strategies to enhance the model’s generalization capability across diverse operational scenarios.

### 3.2 Our conclusion

To address the issue of lost positioning of underwater vehicles, this study proposes a comprehensive mathematical model that integrates three functional modules: positioning, search, and promotion. This model, along with its corresponding algorithm system, offers a systematic approach to predicting the position of underwater vehicles and supporting actual search and rescue operations.

In terms of positioning, the Kalman filtering model is adopted to achieve real-time position prediction, which can effectively handle signal noise and uncertainty, and accurately identify the main sources of uncertainty. The search model constructed based on the Monte Carlo method divides the state of the faulty submersible into four categories. Combined with the positioning information, the initial search deployment point - that is, the planar projection area covering the smallest sample points - is determined, and a spiral search scheme is proposed.

Through reasonable allocation and Python programming implementation, the search probabilities of the underwater vehicle in four scenarios were ultimately determined to be 53.06%, 34.00%, 5.60%, and 25.42% respectively, with significant cumulative search results. This model has good scalability and can be adapted to different sea environments and the needs of multi-underwater vehicle collaborative operations. By adjusting environmental parameters and introducing multi-target tracking algorithms, this model can provide reliable prediction and search support for various sea areas and complex scenarios.

This paper constructs a comprehensive framework system that can handle various emergency scenarios of submersibles. With the proposed model and algorithm, not only has the search and rescue efficiency been significantly improved, but also the safety and economy of the operation have been taken into account. This research achievement is of great significance for enhancing the safety level of underwater exploration and provides a solid reference and in-depth insight for further research in related fields.

## Supporting information

S1 Archive This is the archive containing the source code and figures generated by the code used in the manuscript. This file ensures the reproducibility of the analyses and visualizations presented in the study.
(ZIP)
