# Correction: Search model based on Kalman Filter and Monte Carlo simulation

**DOI:** 10.1371/journal.pone.0346674

**Published:** 2026-04-06

**Authors:** The PLOS One Staff

The second author, Yujing Li, should be noted as having also contributed equally to this work.

The publisher apologizes for the error.
