# Supplementary figures and images for: Search model based on Kalman Filter and Monte Carlo simulation
Source: PLoS One. 2026 Feb 13;21(2):e0339117. doi: 10.1371/journal.pone.0339117 (PMC12904576; doi:10.1371/journal.pone.0339117)

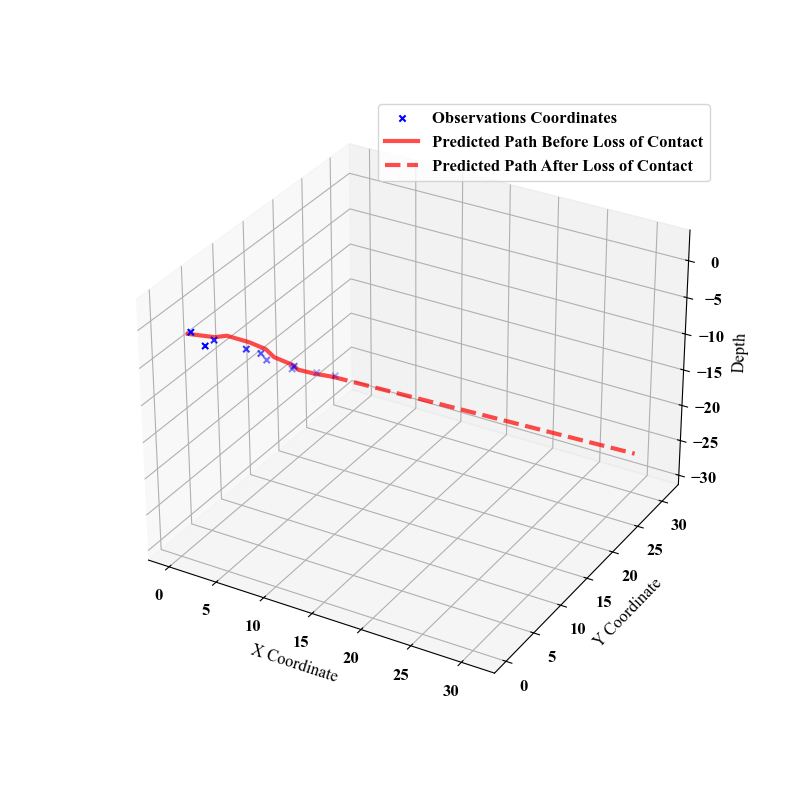

Supplement: S1 Archive — This file ensures the reproducibility of the analyses and visualizations presented in the study. (ZIP) [file pone.0339117.s001.zip › Supporting Materials/Supporting Materials/simulations/figs/karman.png]

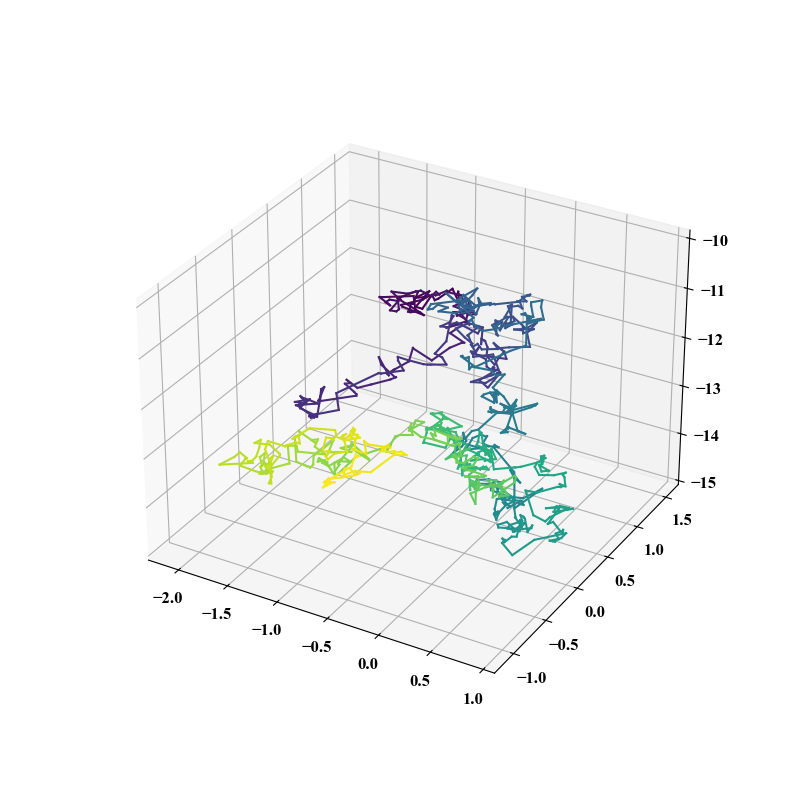

Supplement: S1 Archive — This file ensures the reproducibility of the analyses and visualizations presented in the study. (ZIP) [file pone.0339117.s001.zip › Supporting Materials/Supporting Materials/simulations/figs/random_path.png]

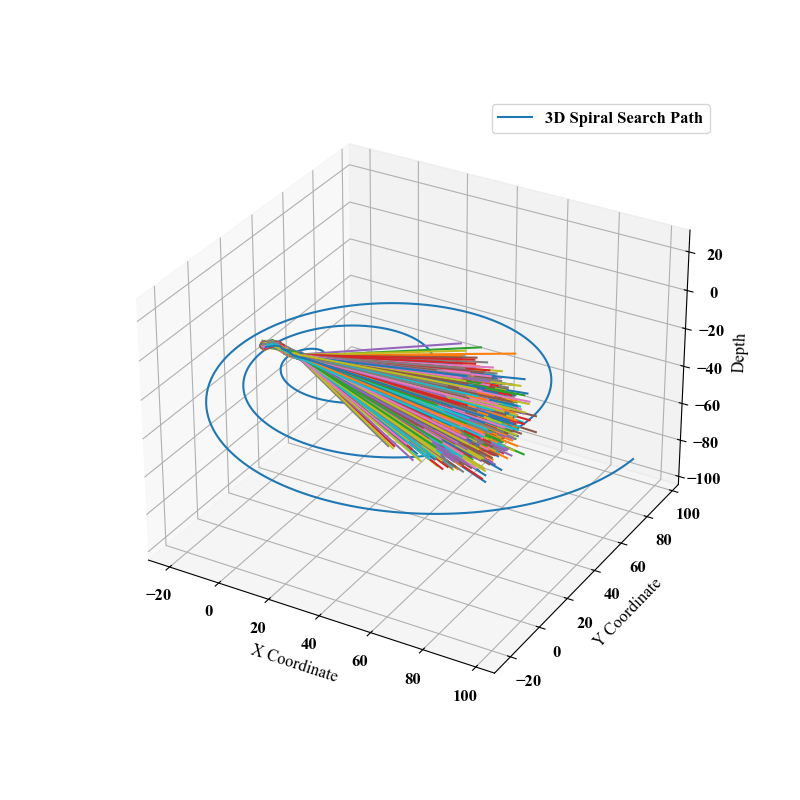

Supplement: S1 Archive — This file ensures the reproducibility of the analyses and visualizations presented in the study. (ZIP) [file pone.0339117.s001.zip › Supporting Materials/Supporting Materials/simulations/figs/simulation1.png]

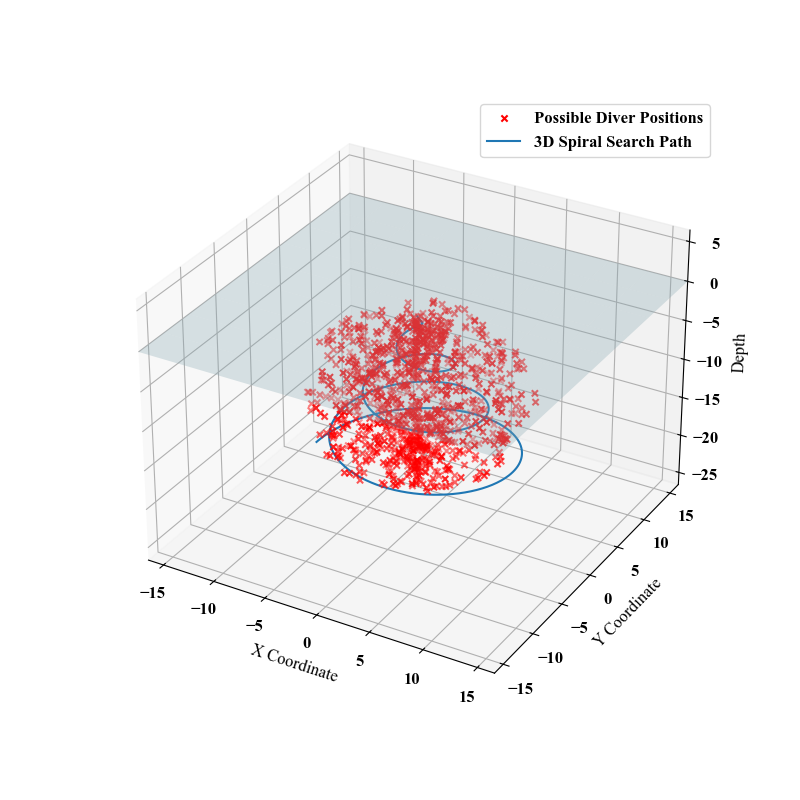

Supplement: S1 Archive — This file ensures the reproducibility of the analyses and visualizations presented in the study. (ZIP) [file pone.0339117.s001.zip › Supporting Materials/Supporting Materials/simulations/figs/simulation3.png]

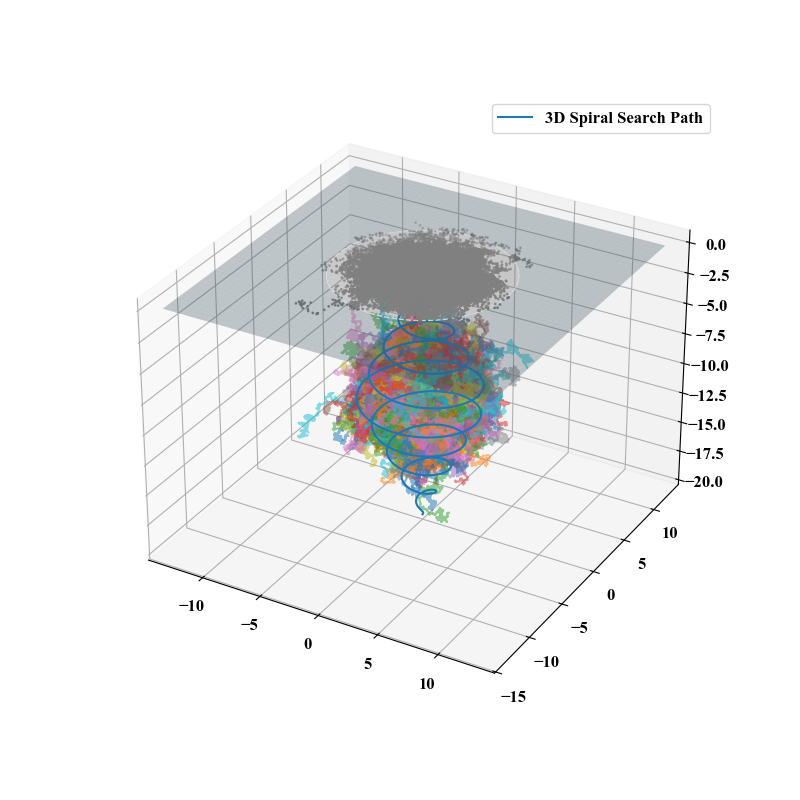

Supplement: S1 Archive — This file ensures the reproducibility of the analyses and visualizations presented in the study. (ZIP) [file pone.0339117.s001.zip › Supporting Materials/Supporting Materials/simulations/figs/simulation4.png]

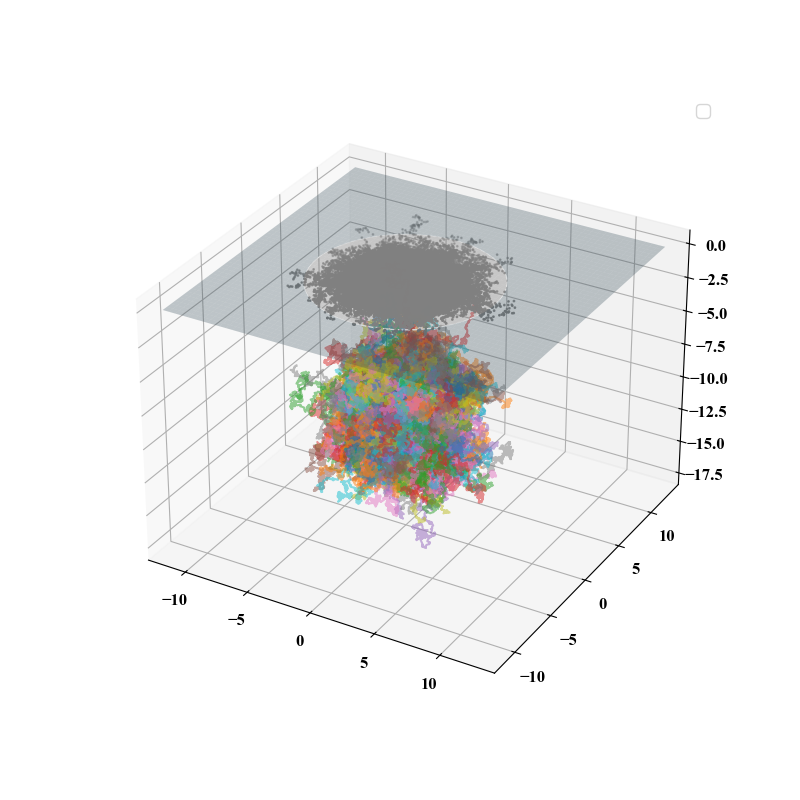

Supplement: S1 Archive — This file ensures the reproducibility of the analyses and visualizations presented in the study. (ZIP) [file pone.0339117.s001.zip › Supporting Materials/Supporting Materials/simulations/figs/┬█╬─═╝.png]
